# Supplementary material for: Biophysical analysis of Plasmodium falciparum Hsp70-Hsp90 organising protein (PfHop) reveals a monomer that is characterised by folded segments connected by flexible linkers
Source: PLoS One. 2020 Apr 28;15(4):e0226657. doi: 10.1371/journal.pone.0226657 (PMC7188212; doi:10.1371/journal.pone.0226657)
Supplement: S1 Fig — (A) SDS-PAGE analysis of the five samples collected from the main peak (lanes 1–5). Ion exchange chromatography of PfHop purification was monitored at 280 nm. Protein that bound to the column was then eluted under NaCl gradient. The five fractions obtained were pooled together for subsequent SEC analysis. (B) SDS-PAGE analysis of several fractions (lanes 1–11) of PfHop obtained by SEC are shown. (C) SDS-PAGE analysis of several fractions (lanes 1–12) of PfHop obtained by SEC are shown. (DOCX) [file pone.0226657.s001.docx]

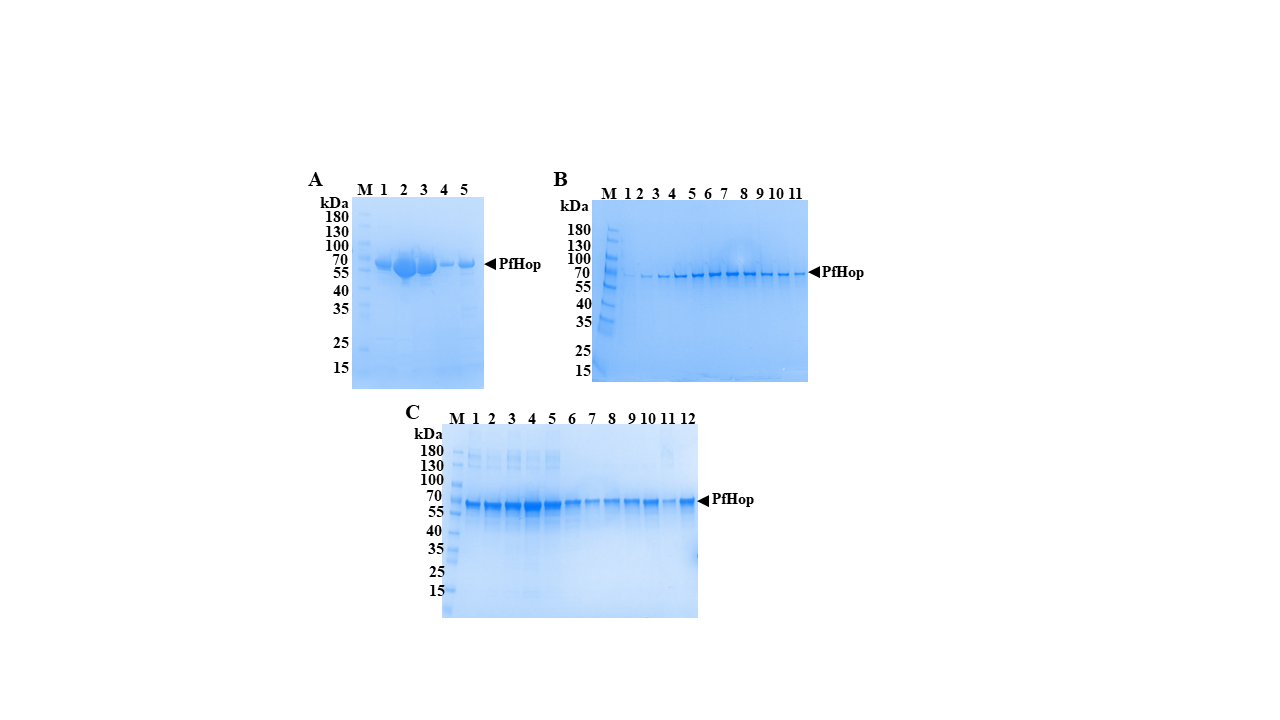


**Figure S1. Purification of PfHop by ion exchange and size exclusion chromatography**

(**A**). SDS-PAGE analysis of the five samples collected from the main peak (lanes 1-5). Ion exchange chromatography of PfHop purification was monitored at 280 nm. Protein that bound to the column was then eluted under NaCl gradient. The five fractions obtained were pooled together for subsequent SEC analysis. (**B**) SDS-PAGE analysis of several fractions (lanes 1-11) of PfHop obtained by SEC are shown**. (C)** SDS-PAGE analysis of additional fractions (lanes 1-12) of PfHop obtained by SEC are shown.
