# Supplementary figures and images for: Biophysical analysis of Plasmodium falciparum Hsp70-Hsp90 organising protein (PfHop) reveals a monomer that is characterised by folded segments connected by flexible linkers
Source: PLoS One. 2020 Apr 28;15(4):e0226657. doi: 10.1371/journal.pone.0226657 (PMC7188212; doi:10.1371/journal.pone.0226657)

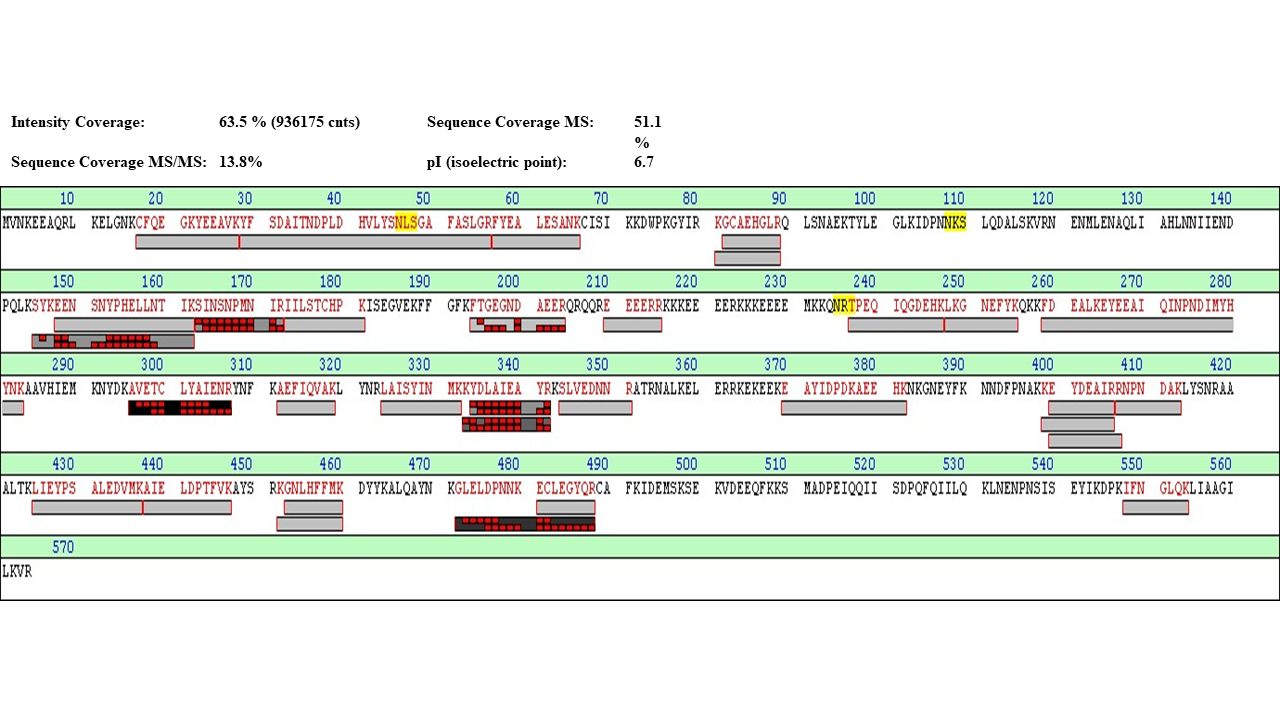


**Figure S2. Mass Spectrometry sequencing data for PfHop**

Supplement: S2 Fig — (DOCX) [file pone.0226657.s002.docx]
