# Supplementary material for: Biophysical analysis of Plasmodium falciparum Hsp70-Hsp90 organising protein (PfHop) reveals a monomer that is characterised by folded segments connected by flexible linkers
Source: PLoS One. 2020 Apr 28;15(4):e0226657. doi: 10.1371/journal.pone.0226657 (PMC7188212; doi:10.1371/journal.pone.0226657)
Supplement: S1 Table — (DOCX) [file pone.0226657.s003.docx]

**Table S1. PfHop secondary structure content**

|  | **Helix** | **Sheet** | **Turn** | **Unordered** | **NMRSD** |
| --- | --- | --- | --- | --- | --- |
| CONTINLL  SP175(175-240) | 77 | 0.9 | 9.2 | 12.9 | 0.235 |
| BeStSel | 77.8 | 0 | 17.8 | 4.4 | 0.059 |
| Phyre | 75 | ND | ND | 4 | **-** |

ND: Not determined
