# Supplementary material for: Biophysical analysis of Plasmodium falciparum Hsp70-Hsp90 organising protein (PfHop) reveals a monomer that is characterised by folded segments connected by flexible linkers
Source: PLoS One. 2020 Apr 28;15(4):e0226657. doi: 10.1371/journal.pone.0226657 (PMC7188212; doi:10.1371/journal.pone.0226657)
Supplement: S1 Raw Images — (PDF) [file pone.0226657.s004.pdf]

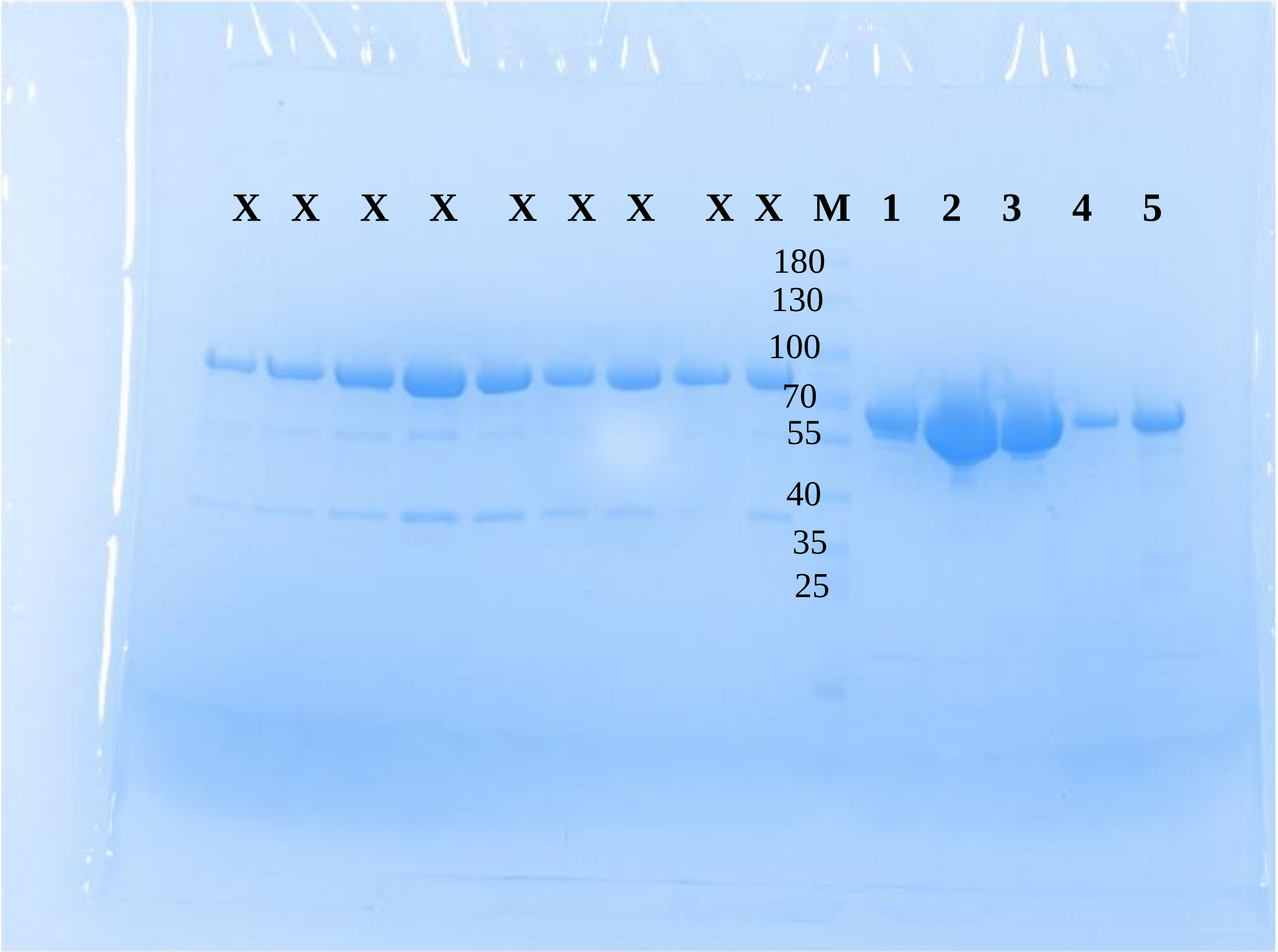

Figure S1A. SDS-PAGE analysis of PfHop fractions obtained after ion exchange chromatography

Lane M: Molecular weight markers (in kDa); Lanes 1-5: PfHop fractions

This image contributed towards supplementary Figure S1A

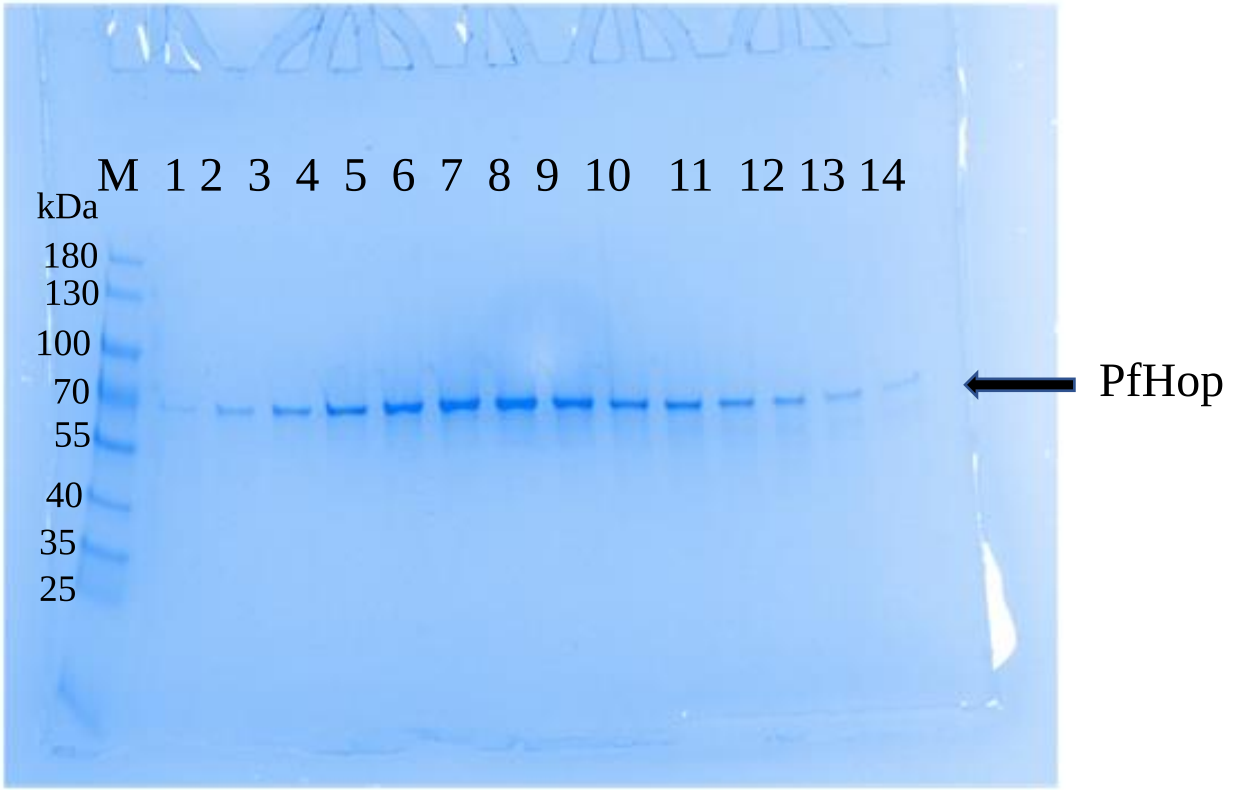

Figure S1B. SDS-PAGE analysis of PfHop fractions obtained by gel filtration
Lane M: Molecular weight markers in kDa; Lanes 1-14: PfHop fractions

This image contributed towards supplementary Figure S1B.

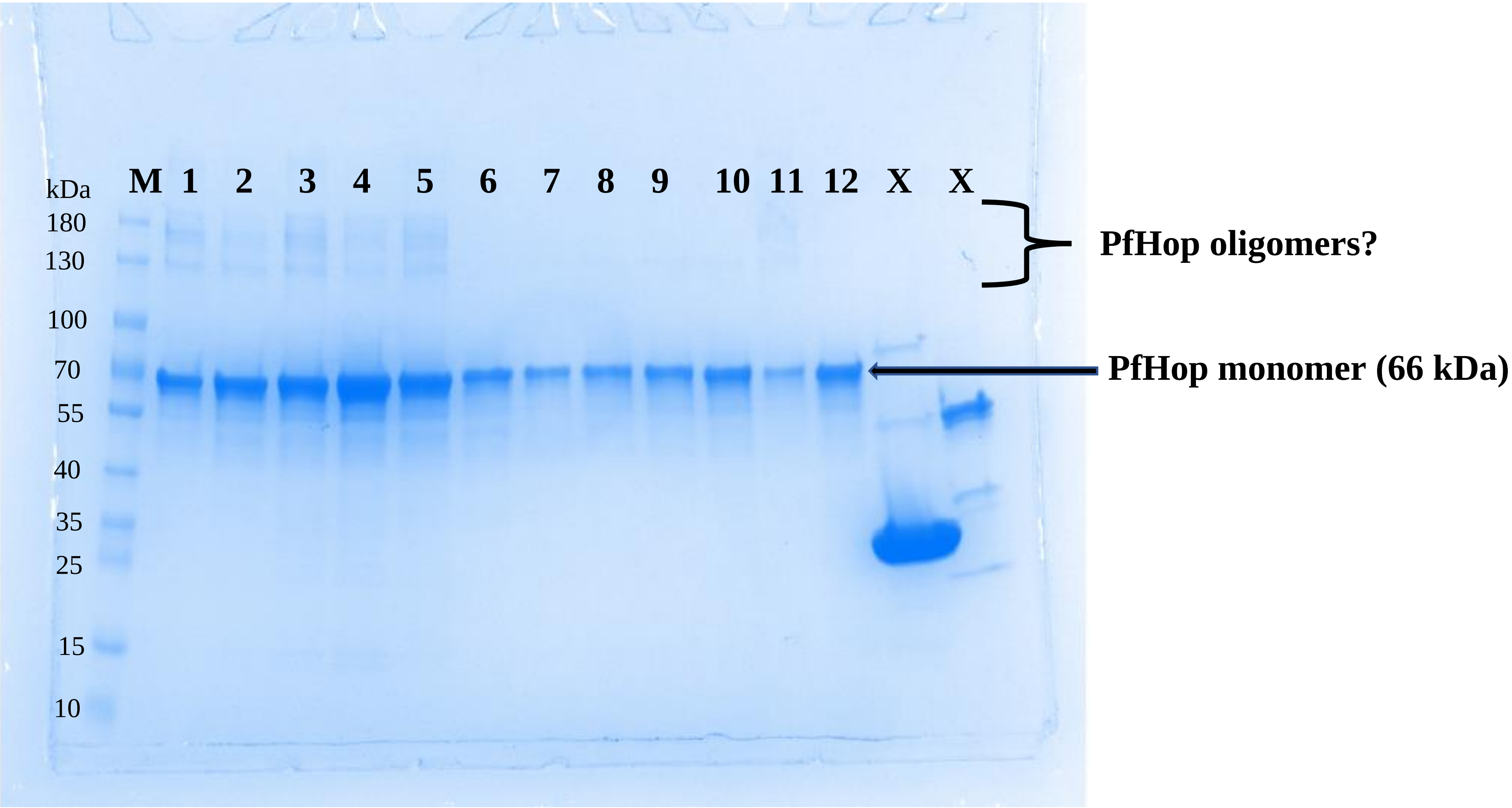

Figure S1C. SDS-PAGE analysis of more PfHop fractions obtained after gel filtration

Lane M: Molecular weight markers (in kDa); lanes 1-12: additional PfHop fractions obtained after gel filtration

This image contributed towards Figure S1C.
